# A systematic literature review to explore lived experiences with phantom limb phenomenon following a lower extremity amputation: a qualitative synthesis

**DOI:** 10.3389/fresc.2025.1667659

**Published:** 2025-09-18

**Authors:** Abdullah Ali H. Alabdullah, Saeed Saad Alyazidi, Ibrahim Ali Asiri, Hussain Saleh Ali, Sarah Abdullah Almutlaqah, Ahmed Saleh Alzahrani, Saeed Abdulrhman Alzahb, Sumaya Abdullah Alasmari, Dejan Nikolic, Natasa Radosavljevic

**Affiliations:** ^1^ Amputation Rehabilitation Unit, Aseer Medical Rehabilitation Center, Aseer Central Hospital, Abha, Saudi Arabia; ^2^ Department of Physical Therapy, Aseer Medical Rehabilitation Center, Aseer Central Hospital, Abha, Saudi Arabia; ^3^ Department of Physical Medicine and Rehabilitation, Aseer Medical Rehabilitation Center, Aseer Central Hospital, Abha, Saudi Arabia; ^4^ Department of Physical Medicine and Rehabilitation, University Children’s Hospital, Belgrade, Serbia; ^5^ Faculty of Medicine, University of Belgrade, Belgrade, Serbia; ^6^ Department of Biomedical Sciences, State University of Novi Pazar, Novi Pazar, Serbia

**Keywords:** phantom limb, lower limb, amputation, experience, qualitative, review

## Abstract

**Objective:**

In this review we aimed to understand better frequent experiences accompanying phantom limb issues from patients’ perspective and accordingly to generate recommendations for clinical practice.

**Methods:**

A systematic literature review approach was utilized and articles meeting the eligibility criteria were critically appraised using the Critical Appraisal Skills Program (CASP). Additionally, a meta-synthesis approach was adopted to combine and analyze the data.

**Results:**

Ten relevant studies were critiqued, key themes were: 1) early Information Provision about phantom Limb Pain (PLP) and Participants’ Satisfaction; 2) the PLP's described characteristics; 3) different Emotions and Psychosocial Issues with PLP; 4) the Impact of the PLP on Performing Daily Activities*;* and 5) the experienced strategies to address the PLP.

**Conclusion:**

The experience of phantom limb varies among individuals with lower limb amputation (LLA); however, for many, PLP significantly affects both physical and psychological well-being, adding an additional burden to the overall experience of amputation. Addressing these challenges should begin with early education, followed by a rehabilitation process that considers individual differences in coping mechanisms. Moreover, patients’ preferences should be prioritized when selecting prosthetic devices and determining the most appropriate treatment strategies for managing PLP.

## 1 Introduction

Lower limb amputation (LLA) is a life-changing incident that impacts many aspects of a patients' life (1), as losing a body part represents a severe disruption of body integrity and is often associated with several challenges (2). It has been acknowledged that individuals with LLA not only need to cope with physical or psycho-social challenges after amputation, but they also need to contend with post-amputation pains (2). These obstacles place a heavy burden on them, and those of their healthcare providers, in succeeding in the rehabilitation process.

Pain following amputation can manifest in many ways. It might relate to the residual limb (i.e., stump pain), which occurs from post-amputation issues (e.g., neuroma) (3), or the pain could exceed the living body part boundaries to appear within the missing limb, which is referred to as phantom limb pain (PLP) (4, 5). PLP could affect individuals differently and has been described variously as, for example, throbbing, twisting, burning, shooting or painful aching arising inside the amputated part's illusory dimensions (4).

The phantom phenomenon appears as pain or just a sensation—so-called phantom limb sensation (PLS)—or both can occur simultaneously whilst being perceived as one issue (5). This overlapping between PLP and PLS could be attributed to their time (following an amputation) and colocation (in the amputated part) (6) but, what is less known is that PLS is not painful (7) and might exist in various forms such as “tight shoes”, foot enlargement sensations or the foot moving to the end of the residual limb, known as a telescoping effect (4, 8). To a certain extent, both issues could influence the rehabilitation process and affect individuals' lives following the amputation.

Several studies have investigated phantom limb syndromes' prevalence rate, and most agreed on the high occurrence of such an issue, despite the different ratios. For example, Stankevicius et al*.* (9), found, in a recent systematic review, that the phantom phenomenon's lifetime prevalence rate was the highest, at 76%–87%. In comparison, the prevalence rate was 49%–93.5% during the one-to-three-month period post-amputation. Although this study was limited to the last five years' articles and failed to justify a strong rationale when excluding old papers, another recent systematic review with a meta-analysis supports the findings by Stankevicius et al. (9). In the study by Limakatso et al. (10), the authors also demonstrated the high rate of phantom limb occurrence, which affects approximately six out of every ten individuals with amputation. The agreement between these two studies and other papers indicates that most post-amputation cases are highly likely to experience phantom limb phenomena at certain points in their lives.

As PLP and PLS are subjective experiences and have a high prevalence and a mysterious nature, it is essential to understand these issues from those experiencing them to enhance the services delivered. This could be achieved through diving in-depth into patients' lived experiences with this phenomenon and generating knowledge from that, which is known as qualitative research (11). Unlike numerical data in quantitative design, qualitative research data are words and descriptions that might be collected using various methods, including interviews or discussing the phenomena in informal sessions known as focus groups (12, 13). Moreover, the results of interviews and focus groups with thematic analysis can produce an interpretation of medical issues such as PLP from the perspectives of those concerned and through professionals' eyes. Although subjectivity is higher in qualitative design that could impact results validity, it exhibits the problem from two different angles (patients view and researchers), which widens the scope of knowledge and makes it popular especially in clinical practices that involve patients in their own care (14, 15).

Literature reviews integrating qualitative studies' findings are greatly needed within patient-driven services. This design can systematically search, break down the findings, examine them, analyze them and draw together the results in one project, which assists in discovering the essential features from several qualitative studies conducted in different settings and cultures (12, 14). As a result, this research design could facilitate the findings' implementation within healthcare practices and maintain services up-to-date (15). That is through accessing one paper rather than many, which saves effort, and the time spent on searching and analyzing data (12).

Despite the recently growing body of literature recognizing qualitative research in healthcare, far too little attention has been paid to qualitative reviews considering phantom limb issues. In fact, the literature search reveals that most reviewers have shown an increased interest in quantitative research that examines the effectiveness of therapeutic interventions to manage the PLP, for example, the systematic review by Wang et al. (16), which evidenced Mirror Therapy effectiveness in reducing PLP by reviewing 11 randomized control trials. Additionally, another recent systematic review by Limakatso et al. (17), supported Graded Motor Imagery intervention as a cost-effective and non-invasive intervention to minimize PLP with limited adverse effects and complications. However, the frequent lived experiences with the phantom phenomena are not yet clear, which is surprising as how to solve a problem effectively without making sense of it first. Therefore, this review aims to interpret and synthesize existing literature on the common experiences of individuals with lower limb amputation (LLA), drawing on patient narratives to highlight key issues, inform clinical practice, and identify future research priorities.

## 2 Methods

The literature search and the meta-synthesis were completed in accordance with the textbook frameworks by Aveyard et al. (12), and Purssell and McCrae (15) for conducting a systematic literature review to systematically identify reliable papers and rigorously synthesize the results.

### 2.1 Searching for studies

The SPIDER tool was adopted to develop the research question that underpins this review, which was defined as “What is the experience of phantom limb phenomenon for individuals with lower limb amputation?”. After building the research question, keywords of the question were identified, and the synonyms were generated by using Mesh terms and Thesaurus.com referred to in Table 1. Further, the SPIDER table (Table 2) was reviewed to determine appropriate eligibility criteria for the search strategy as referred in accordance to guide textbooks (12, 15). The inclusion criteria were: 1) full access articles, 2) english language literature, 3) articles from peer-reviewed journals and 4) qualitative papers. Exclusion criteria were: 1) upper limb amputation and congenital limb deficiency, 2) mix- method and qualitative designs with descriptive analysis.

**Table 1:** Search terms used in the review.

Keywords	Synonyms
Experience*	OR effect*
	OR influence*
	OR outcome*
	OR result*
	OR phenomenon*
	OR impact*
AND	
“Phantom limb pain*”	OR “phantom limb sensation*”
	OR “phantom limb”
	OR PLP
	OR “phantom sensation*”
	OR PLS
	OR “phantom pain*”
	OR “phantom phenomenon*”
	OR “post amputation pain*”
AND	
“lower limb*”	OR “lower extremity*”
	OR leg*
	OR “trans tibia*”
	OR “trans femoral*”
AND	
*Amputation*	OR amputation*
	OR amputee*
	OR “limb loss*”
	OR “limb miss*”
	OR LLA
	OR “limb absence*”
AND	
*Qualitative*	OR interview*
	OR phenomenology*
	OR “lived experience*”
	OR “grounded theory”
	OR “content analysis*”
	OR “thematic analysis*”
	OR narrative*
	OR “unstructured interview*”
	OR “semi-structured interview*”
	OR audio*
	OR tape*
	OR video*
	OR “meta synthesis*”
	OR meta-synthesis*
	OR “focus group*”
	OR “photo voice”
	OR “photo voice”

**Table 2:** SPIDER table.

S	Sample	LLA population
PI	Phenomenon of interest	Phantom limb pain/sensation
D	Design	Qualitative synthesis
E	Evaluation or experience	Patients’ lived experience
R	Research type	A literature review

The first search was undertaken on the Cochrane database, which focuses on high-quality systematic reviews, as advised by Aveyard's (14). The search phrases, Boolean operators, inverted commas, and truncations shown in Table 1 were utilized in this step, and they were afterwards used to search the other databases. After finding no matching systematic reviews in the Cochrane library, a literature search was conducted in January 2025 in four databases: CINAHL, MEDLINE, APA PsycINFO, and AMED. Following that, restrictions were applied and the rationale for which was detailed in Table 3.

**Table 3:** Eligibility criteria for the review.

Criteria	Description	Rationale
Inclusion	Full access articles	Lack of resources enabling access to the unavailable articles.
	English Language literature	The limited time and unavailable resources that are required to translate non-English papers. To decrease the chance of misinterpretation which may impact the validity of the results.
	Peer-reviewed journals	To ensure papers selected for analysis were of good quality that endorse validity of the overall findings and drive to trustworthy conclusions.
	Qualitative papers	Patients’ narrative experiences data can merely be obtained from qualitative papers.
Exclusion	Upper limb amputation and congenital limb deficiency	This review focused on synthesizing frequent experience of PLP amongst LLA. Thereby, Upper limb amputation and congenital limb loss had not been included.
	Mix- method and qualitative designs with descriptive analysis	These designs do not present the participant’ voices as in interviews or focus groups with thematic analysis.
No date limit		It has been found no point in rejecting old articles as the pain experienced in 2025 might not differ from that in the 20th century or before.

**Figure 1:**
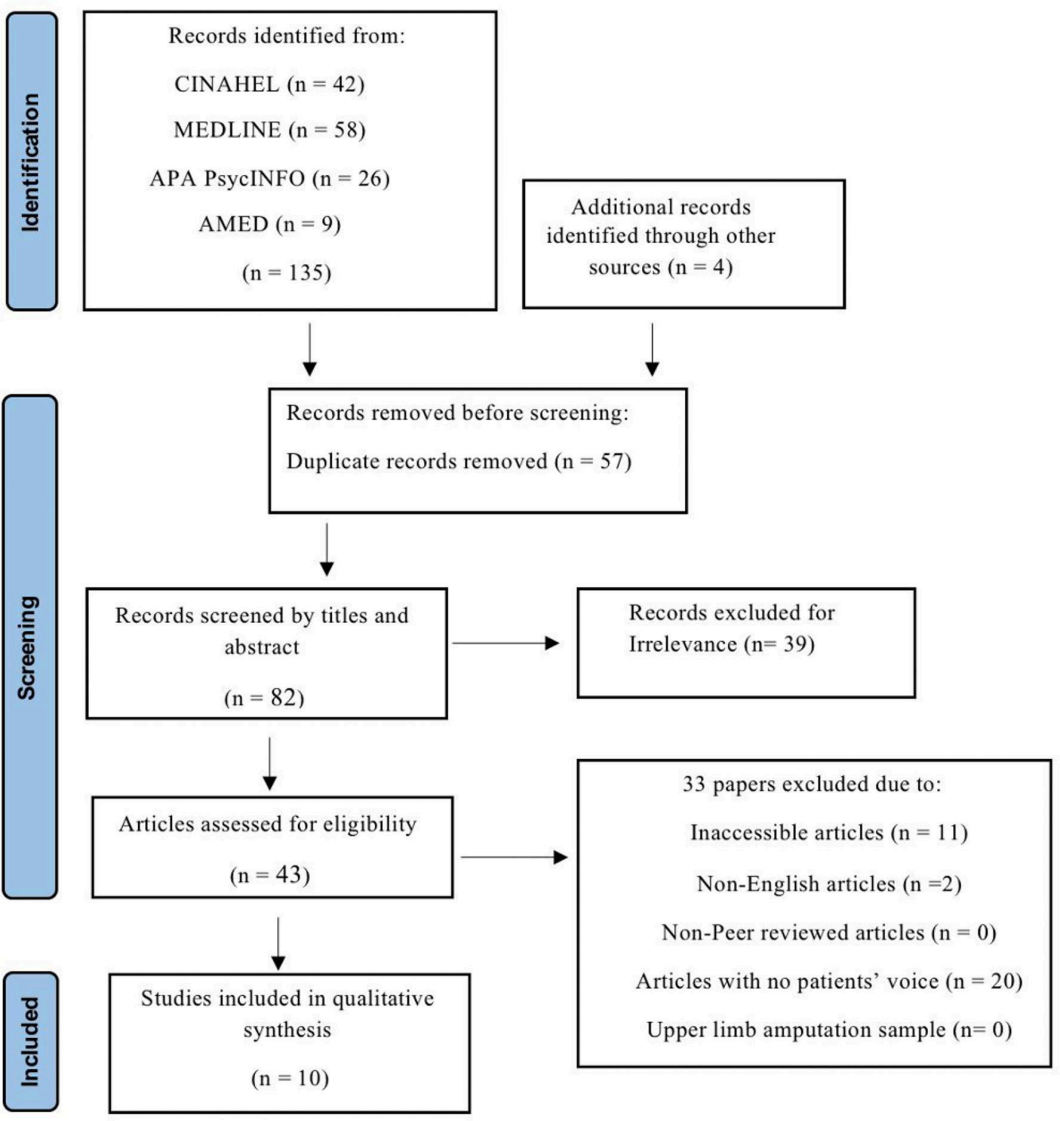
PRISMA diagram for selecting relevant articles.

Returned articles were first verified by reviewing titles and abstracts. If the provided information did not indicate whether work was eligible for inclusion, the full text of the article was then read, and the inclusion and exclusion criteria were also applied. The screening of titles, abstracts and full texts was done by two independent researchers A.A.H.A. and D.N., who were unaware of other researcher screening outcome. In cases where there were disagreements between two researchers, they were included in the discussion process to resolve the one, and in case where agreement was not reached additional researcher (N.R.) was called to propose the recommendation. Search results were recorded in the PRISMA flow chart in Figure 1.

Following this process, ten studies were identified that met all the necessary criteria. For the sake of transparency and comparability, the details of these studies were presented in Table 4.

**Table 4:** The study details included in the systematic review.

Reference	Aim	Participants	Methodology (data collection)	Key Findings/Themes	Limitations (Based on CASP tool appraisal)
Camacho et al. (18)	To explore the lived experiences of support group participants with LLA living with phantom limb and understand the adaptation process postoperatively.	Sample size: *n* = 10; Age: 43–70; Sex: 4 males, 6 females; Level of amputation: AKA: 5, BKA: 5, hip disarticulation: 1. Setting: North of the USA.	A descriptive phenomenological Design: data collected from interview transcriptions. Data analysis involved coding and themes development.	Three key themes were produced: 1. PLP might be Interrupted but does not stop participation. 2. Adapting to PLP allowed for continued participation in meaningful occupations. 3. Education to Promote Self-management of PLP.	Small sample size. Only chronic PLP cases was considered The PLS was not considered The relationship between researchers and participants was not adequately considered
Horne and Paul (19)	To understand the lived experiences of chronic pain support for diabetes-related lower limb amputation patients.	Sample size: *n* = 11; A mean age of 60.82; Sex: 56% male; Level of amputation: LLA due to DM. Setting: South-eastern USA	A qualitative empirical phenomenology design. Data was collected from semi-structured interviews. Data analysis involved coding and theme development	1. Phantom pain is non-treatable A. There was no association of phantom pain with the overall pain experience and B. participants believed that phantom pain could not be helped. 2. Non-empathetic support systems A. Lack of understanding from others and B. analgesics were the only option to treat. 3. Identification of a new normal A. amputation as a choice and B. hope and spirituality.	Small sample size Only diabetic population recruited in this study Only chronic PLP participants recruited (after 6 months) The relationship between researchers and participants was not considered PLS was not considered
Murray (20)	To understand the embodied perceptual experience of successful prosthesis.	Sample size: *n* = 35; Age: 16 years of age or older; Sex: 16 males, 19 females; Level of amputation: LLA: 24, ULA: 3, congenital limb Absence: 8; Setting: The UK	A phenomenological qualitative study design. Data was collected from semi-structured e-mail and face-to-face Interviews. Data analysis involved coding and theme development	The participants in this study described phantom limb phenomena and directly asserted the prosthesis feeling as “part of” them except AKA levels who had that experience temporarily.	Small sample size The ethical issue was not referred to, though the study took place in the UK. The relationship between researchers and participants was not considered. It did not distinguish between the phantom limb pain/sensation.
Nortvedt and Engelsrud (21)	To explore ways in which individuals with phantom pain articulate and understand their pain and their situation	Sample size: *n* = 8; Age: 20–50; Sex: 8 males, 0 females; Setting: (—)	A phenomenological qualitative study design. Data was collected from Interviews.	The participants described their pain using vivid and disturbing metaphors, such as feeling invaded by insects or having their skin burned and torn away. These expressions reflect how pain affects their sense of self and their connection to others. Phantom limb pain (PLP) deeply impacted their lives, altering their relationship with their body, other people, and the world around them. Many saw the pain as a haunting reminder of their once whole and functional body, creating a painful contrast between their past and present selves.	Small sample size. This study only conducted on male participants Along with LLA participants, this study recruited participants with a total lesion of the spinal cord and combined both narrative experience in the results, which made extracting data that related to LLA challenging. The relationship between researchers and participants was not adequately considered
Devan et al. (22)	To examine the perceptions of people with a lower limb amputation as to important factors contributing to their low back pain	Sample size: *n* = 11; age: 18–70 years; 8 males, 3 females; Level of amputation: AKA: 3, BKA: 8; Setting: New Zealand	A qualitative study design. Data was collected from Semi-structured interviews. Interviews were conducted in (three focus groups and two individual interviews	The participants stated that they experienced more incidences of phantom limb pain when they had LBP. The participants also complained that severe PLP impacted their walking with the prosthesis as well as their sleeping.	Small sample size The relationship between researchers and participants was not considered
Mortimer et al. (23)	To explore patients’ experiences of phantom pain, their perceptions of the current information received on phantom sensation and pain, and their views on improving this information.	Sample size: *n* = 31; age: adults aged less than 75 years; 18 males, 13 females; Setting: Scotland	A qualitative study design. Data was collected from focus groups. Each participant in this study took part in one of seven focus groups.	The status, severity, and nature of phantom limb pain (PLP) varied widely among individuals. Many felt helpless due to a lack of understanding about the PLP. Experiences with healthcare professionals’ guidance also differed—some received helpful information, while others got little or none. Patients strongly felt that clear, verbal, one-on-one explanations should be provided before or shortly after amputation.	Small sample size The relationship between researchers and participants was not considered
Trevelyan et al. (24)	To explore LLAs’ lived experiences of PLP, to understand how PLP impacts quality of life and to determine whether amputees feel they are provided with adequate information about PLP.	Sample size: *n* = 15; 12 males, 3 females; Level of amputation: AKA: 6, BKA: 8, TKA: 1; Setting: The UK	A qualitative study design (semi-structured interviews). 1–3 months post-surgery with past or current experience of PLP were interviewed once about their experience of PLP. Interviews were audio-recorded, transcribed verbatim and analyzed using Framework Analysis. Interviews were conducted in an amputee rehabilitation unit in London.	Six key themes were identified during analysis. Three were related to PLP and are reported on in this article; 1) real and physical phantoms, 2) living with a phantom and 3) being informed. 4) PLP had numerous painful qualities. 5)The phantom felt real, with kinetic and kinesthetic properties. PLP had multiple meanings to amputees, 6) was considered a reminder of circumstances and could affect quality of life. Information provided about PLP was inadequate.	Small sample size The relationship between researchers and participants was not considered
Bragaru et al. (25)	To identify the barriers and facilitators that influence participation in sports for individuals with LLA.	Sample size: *n* = 26; 19 males, 7 females; Level of amputation: HD: 1, AKA: 11, BKA: 6, TKA:6, AD: 2; Setting: Netherlands	A qualitative study design. Data was collected from semi-structured interviews. It follows thematic analysis to analyze data	Phantom pain represented a motivator to participate in sports. Most of the athletes who experienced (phantom) pain mentioned that: “it. decreased in intensity or even completely disappeared”. For some interviewees phantom pain acted as a barrier: “Because I have a low pain threshold, I can’t participate in sports adequately”	Small sample size The relationship between researchers and participants was not adequately considered
Björkman et al. (26)	To understand the patients’ personal experience of phantom phenomena	Sample size: *n* = 28; age: 18–80 years; 12 males, 16 females; LLA: 20, mastectomy:8 Setting: Sweden	A qualitative study design. Data was collected from interviews using focused, open-ended questions.	In many cases, phantom sensations and kinetic perceptions were more troubling than the PLP itself. Patients who had mastectomies described their phantom experiences differently, often struggling to locate or describe the phantom breast. PLP often began as intense pain but tended to decrease over time. Patients used vivid metaphors like “boiling water” or “someone digging into your Achilles tendon” to describe the pain, and many expressed strong negative emotions, such as sadness and distress.	Small sample size The relationship between researchers and participants was not considered
Rich et al. (27)	To understand the PLP experience and patients’ familiarity with management, phone interviews were conducted at the Minneapolis Veterans Affairs Regional Amputation Center in Veterans with amputations.	50 Veteran participants (average age 66, 96% male) with lower limb amputation were recruited	A qualitative cross-sectional cohort study design. Data was collected from phone semi-structured, open-ended interviews at the Minneapolis Veterans Affairs Regional Amputation Center in Veterans with amputations.	They identified three core themes from the qualitative interviews including 1) high variability in the experience of PLP, 2) acceptance and resilience, and 3) PLP treatment perceptions. The majority of participants reported trying common non-drug treatments with none endorsed consistently as highly effective.	The majority of participants were male (96%) and white (92%). The study mostly looked at veterans with lower limb amputations, which were most likely caused by traumatic injuries. Therefore, the results may not be generalized to individuals with amputations from other causes, or to females or individuals from different racial backgrounds. The relationship between researchers and participants was not considered The ethical issue was not referred to, though the study took place in the USA.

HD, hip disarticulation; BKA, below knee amputation; AKA, above knee amputation; TKA, through knee amputation; AD, Ankle disarticulation; LLA, lower limb amputation; PLP, phantom limb pain; PLS, phantom limb sensation; ULA, upper limb amputation; LBP, lower back pain; DM, diabetes mellitus; UK, The United Kingdom; USA, The United States of America.

### 2.2 Appraising the included studies' quality

**Figure 2:**
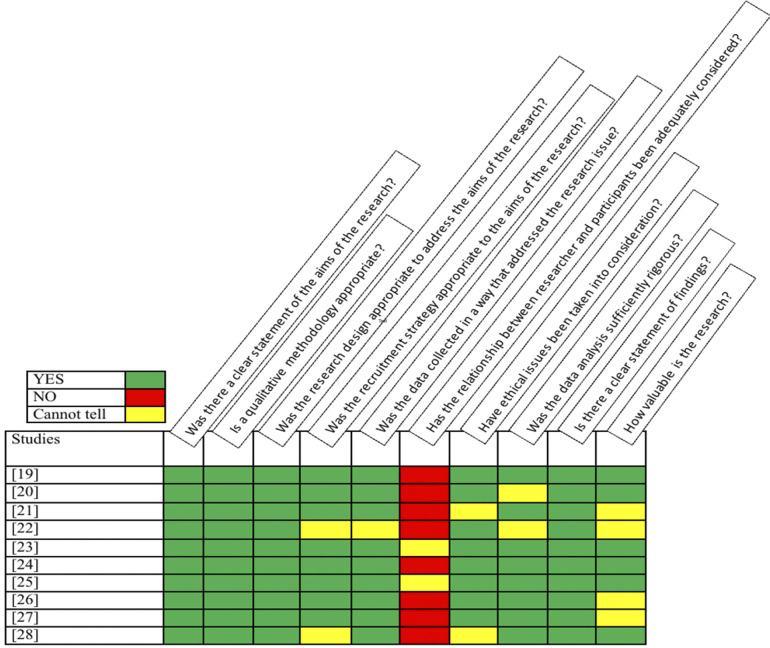
The CASP tool critical appraisal summary.

The Critical Appraisal Skills Program (CASP) tool for qualitative studies was used to assess the quality of each included paper and the results are summarized in Figure 2. This tool is widely used and recognized as a valid critical appraisal checklist that has been recommended by the National Institute for Health and Care Excellence (NICE) (28) and recognized in the guidelines (12, 15). The CASP tool was used to assess the strengths and weaknesses of studies in ten areas that are considered relevant for evaluating qualitative research, such as the relevance of the studies' design to answering the research question and their ethical considerations. This process was performed by A.A.H.A., N.R., S.S.A., I.A.A., S.A.A., S.A., and D.N. during consensus meetings, in which each author independently applied the CASP tool. The results were then discussed and collaboratively revised into a unified version summarized in Figure 2. Further details of the main agreed-upon limitations are presented in Table 4.

The importance of ethical issues in human-related research was recognized. Therefore, the articles conducted outside the United Kingdom (UK) or The United States of America (USA) and did not declare approval from ethical committees or institutions were excluded as this was considered to be a flaw that indicates their results' weaknesses.

### 2.3 Analyzing and synthesizing the included studies

**Figure 3:**
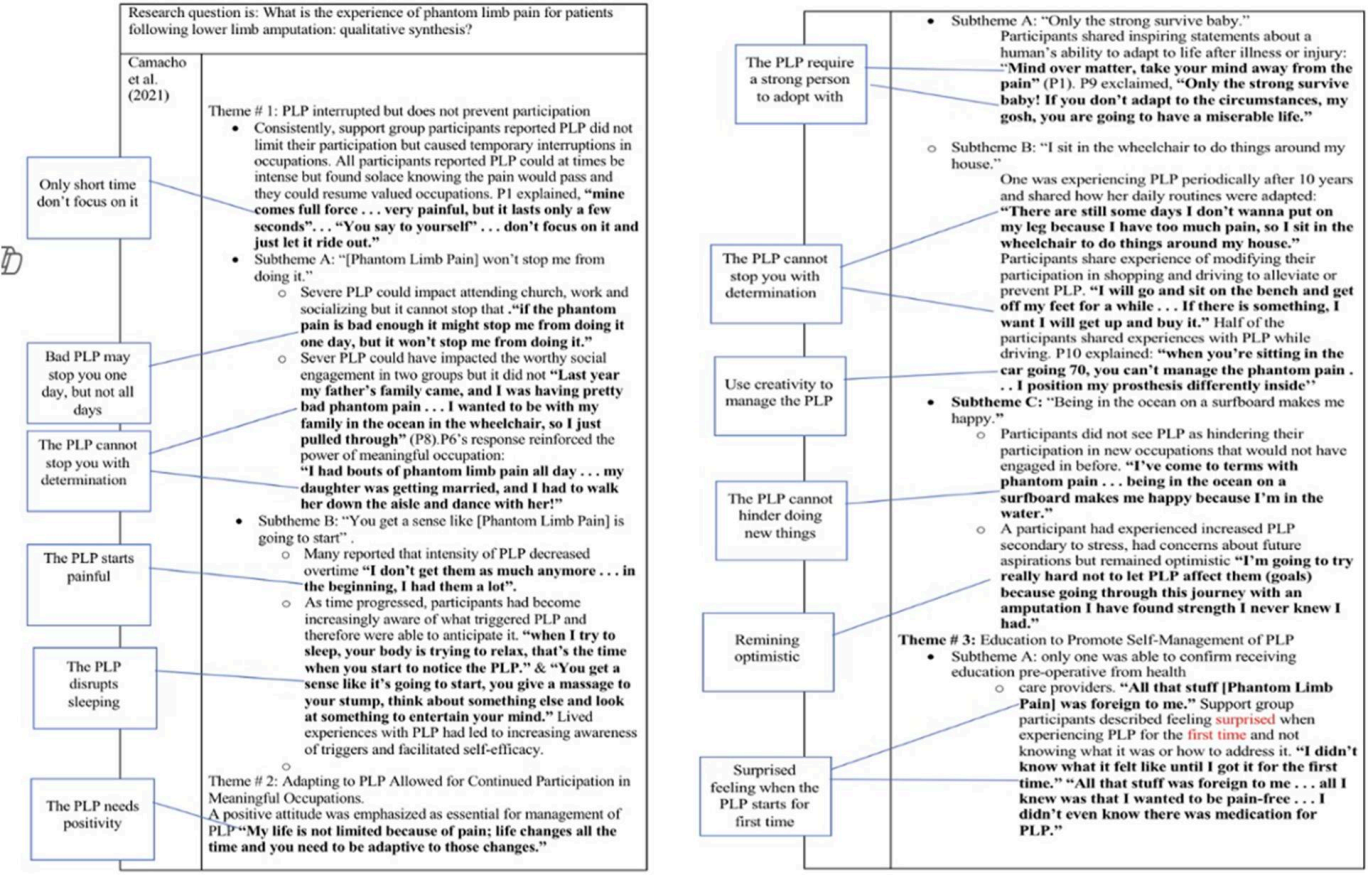
**(A–D)** A summary of analyzing and synthesizing process. **(A)** An example of how coding was done. **(B)** An example of how codes and quotations were extracted and compiled in a separate table. **(C)** An example of how codes were grouped according to discrete ideas resulted from comparison between codes and how themes were generated. Each circle's color represents a study. **(D)** An example of how codes and themes were organized in a table.

The data extraction to collect relevant quotes and its synthesis were based on Murray and Forshaw's (14) approach and the guidebooks (12, 15). First, quotations relevant to the research question were identified from the results' sections in each article. Second, for each article, all citations and phrases relevant to this review's question were coded, extracted and compiled in a separate table. After this process, the codes were grouped and labelled according to the discrete concepts that resulted from a constant comparison between the codes within and between each article. Over these stages, the authors' names were maintained by referencing each quote and using colors to identify their origin. Themes were then generated based on the new sets of codes and linked quotes, which were subsequently organized in another table. Finally, the resulting themes were examined, with a focus on how well they capture the original themes within each study and their relevance to the research question. The analysis and synthesis procedures are summarized in Figure 3.

## 3 Results

In this systematic review, 10 studies were included from 2002 to 2023. A total number of patients in analyzed studies was 225. Nine studies evaluated both genders, while one study evaluated male population.

Five concepts were identified from meta-synthesis, and each theme was presented alongside indicative quotes from the original papers' participants.

### 3.1 Early information provision about PLP and participants' satisfaction

This theme was addressed in three papers (18, 23, 24). On one hand, few participants declared they received early information about PLP which was a helpful experience that made them satisfied with the service provided: “the therapist always answered my questions and gave me good information that was so helpful because it was all new and I had no idea” (18).

On the other hand, the majority expressed dissatisfaction with the PLP information provision. This was attributed to; firstly, the timing of the information being given in relation to experiencing it: “they didn’t tell you early enough, you experienced it especially the foot still being there and they then try and get you to stand up. I think they should be telling you that very soon…that you have to be very careful” (23). Secondly, the source of the knowledge as most information was not delivered by healthcare providers unlike what had been expected: “the only people who have explained it to me are other patients. But no doctors or nothing mentioned anything to me about the pain (PLP)” (24).

Although the majority of participants favored information pre-operatively, others conversely thought it could be alarming: “You’d probably alarm people” (23) and cannot be perceived because of dominant thinking about amputation at this time: “I think after it, because before the thought of having it off is uppermost in your mind” (23).

### 3.2 The PLP's described characteristics

This was addressed in seven studies (18, 21–24, 26, 27). As pain is a subjective experience, the participants expressed various PLP's characteristics, with some using aggressive language to articulate the sensations e.g., a feeling of “gripping sticking a needle in your leg” (23). Some used metaphors to express their pain “like boiling water” (26) and like “someone is digging into your Achilles tendon” (26), “It (PLP) feels like glowing red-hot rebar that I stepped on or 100,000 needles or knives” (27), while the rest merely indicated that they experienced odd sensations. However, painful “itching” was the frequent type mentioned in the participants' descriptions: “and it itches! But it's very difficult to explain. It's as if I am lying in a nest of insects, and they’re constantly crawling not only outside but inside my body” (21).

In addition, there were clear variations between the experiences regarding the PLP's intensity over time. Some amputees had severe pain in the beginning, which decreased with time: “I don’t get them as much anymore … in the beginning, I had them a lot” (18). Others experienced the opposite scenario: “Now the first 20 years for me when I was an amputee, I never had any and certainly when you get older, you’re more prone to getting it (PLP)…” (22).

### 3.3 Different emotions and psychosocial issues with PLP

This theme was discussed in six papers in which patients shared various emotions relating to their PLP (18, 19, 21, 23, 24, 26). Initially, participants had surprising reactions because they felt their PLP as real physical pain, which had not been expected: “I was surprised how real the pain seemed, it was real and now I realize, but then I was surprised at the level of pain particularly in the toes” (23). Over time, some expressed negative emotions towards their PLP, such as frustration that they have lost a limb and having to cope with that, but there was the PLP that constantly reminds them of this loss: “But it's also a reminder. The phantom pain reminds me that my leg is gone forever, so I don’t get any opportunity to forget that’… ‘and that troubles me…” (21). Furthermore, intense PLP drove them to face negative psychological consequences, such as depression: “I’m very sad.” (26)—or mood swings—“Cause you are in pain … you become irrational, you become snappy, you become less patient with people even though you may not have a reason for it” (24)—or having suicidal thoughts—“Um and if I was on the second floor of a building I’d probably want to jump out of the window because the pain just gets so much you can’t cope with it” (24).

Conversely, the PLP was viewed positively by others when it was considered better than pre-amputation pains, when the patients liked having sensations in the missing limb, when it was perceived as good pain and when the amputee was glad just to be alive: “It (PLP) makes me feel glad I’m alive really … As long as you are feeling something!” (24).

### 3.4 The impact of PLP on performing daily activities

Seven papers discussed this theme (18, 21–25, 27), some participants complained that their severe PLP disrupted them in performing daily activities, such as walking with prostheses: “It (PLP) seems to be more, more involved, it can actually make your walking hard to do cause it's there all the time in the socket and ah it's almost a numbing effect” (22). Others also highlighted that severe PLP causes distraction, which hinders performing activities that require concentration such as, eating, reading or driving: “Right now, the pain is so terrible that I just forget. The pain steals all of my concentration so I don’t remember what I read” (21).

Participants also stated that the most common time for experiencing PLP was when relaxing and lying on the bed, which impacts their resting and disturb their sleeping: “I’ve had nights where I haven’t slept so you just can’t ‘cause it just grabs you all the time’” (22).

On the other hand, the participants manifested the silver lining of having PLS that had a positive impact on their prosthetic experience thus, on their walking. The PLS was found useful in enhancing prostheses' embodiment (making it feel part of their body) and provides kinesthetic sensation in a number of the participants' experiences: “It is certainly nice to still feel the (phantom) foot. Primarily, it facilitates the use of the prosthesis because I don’t feel as anything is really missing. So, my prosthesis is “natural” (20), another participant states: “it's just there, it's trying to tell me where my foot is, which is good. So, it's good pain if that makes sense” (24).

### 3.5 The experienced strategies to address the PLP

In six of the included studies (18–20, 23, 24, 27), participants shared some creative non-medical strategies that worked well to control their PLP, such as using household goods, rubbing the residual limb, distracting themselves or finding enjoyment in their life to forget the pain: “If it (PLP) happens at night, I will get up and distract myself with TV or the computer” (27), “I was cleaning out the refrigerator and all of a sudden I got this pain. I placed it inside the refrigerator and the cold and leaning helped” (18) another participant states: “you give a massage to your stump, think about something else and look at something to entertain your mind” (18).

Despite severe PLP potentially causing temporary disruption to activities, determination and adopting a positive attitude towards their situation have assisted some participants to cope positively with the pain and live their lives: “if the phantom pain is bad enough it might stop me from doing it (attending church) one day, but it won’t stop me from doing it” (18). The participants also recognized the necessity of adjusting themself to the new situation with pain to survive: “Only the strong survive baby! If you don’t adapt to the circumstances, my gosh, you are going to have a miserable life.” (18).

Interestingly, some participants shared that they did not favor taking drugs to manage their PLP as they found that their own strategies control their PLP better within a short term than prescribed medicines: “I’m not one to take medicine … I like to take myself to like the beach … my mind is being taken away from the pain” (18).

## 4 Discussion

The review aimed to gain insights into the patients' lived experiences with phantom limb issues to make recommendations for clinical practices. The themes displayed different experiences; nonetheless, this section will discuss the interesting points.

The participants revealed a lack of consistency in providing pre-amputation education by professionals, even though these sessions were recommended in guidelines such as the British Association of Chartered Physiotherapists in Amputee Rehabilitation (BACPAR) for physiotherapists (29). Unfortunately, this absence of early education in their experiences contributed to most of their dissatisfaction with the PLP management services. These reactions could be explained via the study by Ostler et al. (30) who asserts that patients' decision making in post-amputation rehabilitation could depend on the early education of what to expect before it occurs. Such education can enable individuals with amputation to make informed decisions during rehabilitation, (such as, PLP's treatment methods) and ignoring early education would lead patients to either passively accept what others suggest due to a lack of knowledge or to conceal their experiences out of fear of being perceived as mentally ill.

Besides, few participants argued that providing early information is required, but it needs to be delivered immediately after amputation surgery (instead of before) to avoid alarming patients while making critical decisions about their lives. In contrast, Gallagher et al. (31) found that not having pre-amputation education was positively associated with post-amputation PLP occurrence in a study representing 104 participants. That led them to recommend these counseling sessions as a kind of support to address stresses, avoidance and exaggerating reactions towards PLP preoperatively. Similarly, Mareboina et al. (32) explore a human-centered approach to optimizing the management of phantom limb pain, concluding that early counseling significantly enhances treatment outcomes.

The evidence highlights the critical importance of early education on PLP, as it has the potential to significantly enhance patients' rehabilitation outcomes. Accordingly, Rehabilitation healthcare practitioners should prioritize the provision of such educational interventions, ensuring they are delivered in a timely, sensitive, and patient-centered manner that aligns with individual preferences.

The reviewed studies reported various psychological issues associated with PLP, but the variation between participants' coping styles with limb loss and the PLP role was interesting. Although the majority expressed that their hopeful outlook on life and faith assisted them in positive coping and in controlling this pain, few were frustrated at their inability to adjust to the new situation after amputation blaming their PLP for constantly reminding them of limb loss. This variation gives value to Pucher et al. (33) results, who found that adjustment to amputation is a highly individual process and asserted that following a positive coping strategy is critical in a positive adjustment to amputation. Further, they illustrated that those who cope better with the loss suffer less from PLP. Another study conducted by Unwin et al. (34) on 99 patients emphasized the psychosocial variables’ role in the general adjustment. The study uncovered that hope leads to a positive mood and that both hope for a better future and appropriate social support contribute to positive adjustment to LLA. Thus, PLP existence could be a factor affecting adjustment to amputation, and because of the different coping styles between patients, it is necessary for health care providers to identify what hinders the coping process (especially, PLP). It can be via listening more to patients' issues during the rehabilitation period then helping them develop the mentioned coping strategies. Additionally, psychological interventions such as cognitive behavioral therapy (CBT) could be utilized when teaching ways to cope with the loss and develop the attributes of those who find positive meaning from PLP experience to facilitate adjustment to amputation, as suggested by Oaksford et al. (35) and Murray and Forshaw (14).

The participants recognized the lived incorporation between their artificial and phantom limbs. For them, the PLS allowed the prosthesis functioning as an extension of their “skeletal system” and to feel the prosthesis as a continuation of their bone. This embodiment between prosthetic and phantom limbs was examined in a study by Bekrater-Bodmann (36) on 148 participants. It was found that the co-location of phantom and artificial limbs contributes to prostheses' embodiment, particularly in LLA cases. The author attributes this phenomenon to the impact of PLS, which seem to cognitively transfer their inherent vividness to the prosthetic limb, thereby facilitating its incorporation as an extension of the body. Crawford (5) agreed with this view, arguing that phantoms have amenable characteristics that facilitate technological conjoining with the prosthesis, which could explain this relationship. However, this is not to suggest that all prosthesis users will experience positive embodiment associated with phantom limb sensations (PLS), as such experiences are inherently subjective. Indeed, in one of the reviewed studies (20), some participants reported perceiving their prostheses merely as functional tools rather than as integrated parts of their body. It is important to note that the embodiment of prostheses can also be influenced by various factors, including psychological aspects in body image perception, as well as the acceptance of and satisfaction with the prosthesis in its functional role, as highlighted in the study by Bekrater-Bodmann (37). Thus, PLS might enhance the prosthetic experience by incorporating the prosthesis into the users' bodies. However, a rehabilitation team involving physiotherapists, psychologists, and prosthetists should recognize the other mentioned factors as well, involve patients when choosing their prostheses, and encourage them to early train with prostheses, to facilitate prostheses acceptance.

Participants in the reviewed studies expressed a less favor to pharmacological treatment options and greater preference for non-pharmacological self-treatment strategies due to satisfying results of these strategies that served their need, which was PLP reduction within a short period. Self-distraction and residual limb massage techniques were often used among these individual strategies and were expressed to be effective in controlling severe PLP. Ketz's (38) study investigated the effectiveness of these two individual techniques compared to standard pharmacological methods (e.g., gabapentin and opioid analgesics) in a retrospective descriptive questionnaire for 30 amputation cases. The study found that participants' descriptions of their PLP and the perceived relief from treatments varied greatly with both methods, leading the author to support the concept that no single treatment technique works for all PLP cases. This variation lends weight to the suggestion from Oleksak (39), who also found similar variations between both treatment approaches and, thus, suggested selecting an appropriate treatment on an individual basis. This patient-centered treatment approach could contribute to a positive experience as healthcare providers might have varying views on what patients consider as a successful outcome regarding PLP treatment (40). Therefore, involving patients in deciding the best PLP treatment option is required to fulfil their expectations from post-amputation rehabilitation. This suggestion can be implemented in individual sessions in which professionals exhibit treatment options, actively listen to patients’ preferences, and subsequently, co-decide with patients the appropriate treatment technique.

## 5 Strengths and limitations

This review followed a systematic approach informed by expert guidance and relevant literature. It identified key concepts shaped by participant characteristics (e.g., age, sex, type, cause, and timing of LLA) and study settings (e.g., recruitment methods and country), enhancing the relevance of findings for diverse healthcare contexts.

Nonetheless, certain limitations must be acknowledged. Non-English studies were excluded. While interpretations were also limited by how data were presented in the original studies—a common limitation in meta-synthesis (41)—regular discussion with co-authors from different rehabilitation backgrounds helped mitigate potential bias. Another limitation refers to the time of the study conduction, considering the fact of rapidly evolving nature of research in rehabilitation and phantom limb phenomena.

## 6 Implications

The qualitative synthesis of ten studies offers a comprehensive view of common themes in the experience of phantom limb phenomena. These findings provide a strong basis for future research on shared aspects of PLP. Despite noted limitations, the review highlights the importance of patient-centered care and offers practical insights for improving rehabilitation services. Future research should examine patients' perspectives on their PLP improvement in relation to time since amputation and its causes.

### 6.1 Implications for rehabilitation

1. Phantom limb pain can be a particularly challenging experience due to its intense and often aggressive nature, as well as the resulting physical and psychosocial difficulties it imposes. Qualitative research on subjective experiences, such as phantom limb pain (PLP), is essential for informing the design and delivery of rehabilitation services that effectively address the needs of this patient population.
2. The findings of this literature review suggest that effectively addressing phantom limb experiences in individuals with lower limb amputation (LLA) requires increased emphasis on educational interventions and the adoption of a patient-centered approach by healthcare providers.

## 7 Conclusions

The phantom limb experience varies among individuals with lower limb amputation (LLA), but most find it distressing due to its intensity and impact on daily life and mental health. A synthesis of patient experiences underscores the need for improved amputation rehabilitation services. Early education on phantom limb pain (PLP) and treatment options—ideally before or soon after surgery—can support informed rehabilitation decisions. Healthcare providers should address patient concerns, offer psychological support, and encourage strategies that aid adjustment and reduce their PLP. Involving patients in prosthesis selection and promoting early use may also enhance prosthesis embodiment by harnessing phantom limb sensations (PLS). Implementing these strategies within a patient-centered framework can significantly enhance the rehabilitation experience and outcomes for individuals with PLP.

## Data Availability

The original contributions presented in the study are included in the article/Supplementary Material, further inquiries can be directed to the corresponding author.
